# Clinical Manifestations of Non-Congenital CMV Infection in Infants and Immunocompetent Children: Review of Cases from the Past Decade

**DOI:** 10.3390/microorganisms13040772

**Published:** 2025-03-28

**Authors:** Chryssoula Tzialla, Serena Salomè, Vito Mondì

**Affiliations:** 1 Neonatal and Pediatric Unit, Polo Ospedaliero Oltrepò, ASST Pavia, 27058 Voghera, Italy; 2 Division of Neonatology, Department of Translational Medical Sciences, University of Naples Federico II, 80138 Naples, Italy; serena.salome@unina.it; 3 Neonatology and Neonatal Intensive Care Unit, AO San Giovanni-Addolorata, 00184 Rome, Italy; vmondi@hsangiovanni.roma.it

**Keywords:** cytomegalovirus, immunocompetent, postnatal, primary, infant, children

## Abstract

Cytomegalovirus (CMV), the largest of the herpes viruses, is a widespread virus that commonly infects people of all ages. CMV can cause a spectrum of clinical manifestations, ranging from asymptomatic infection to severe disease, particularly in immunocompromised hosts. However, postnatal and acquired CMV infections in immunocompetent children remain under-documented in the literature. In this review, we examine studies published over the past decade to explore the clinical manifestations of CMV infections in the pediatric population, focusing on the variety of symptoms and the severity with which the infection can present. Papers published between 1 January 2014 and 2 December 2024 were selected from PubMed/MEDLINE, Embase, Scopus, and Web of Science. The search was conducted using the following keywords: “cytomegalovirus”, “child”, and “immunocompetent”. The target population ranged from 0 to 17 years of age, with congenital and perinatal infections excluded. Despite the clinical significance of CMV in immunocompetent infants and children, there is a lack of consensus on the use and duration of antiviral therapy. This article aims to enhance clinicians’ understanding of the various presentations of CMV infection in immunocompetent children, with the goal of facilitating earlier diagnosis and appropriate management. The reviewed papers indicated that postnatal CMV results in liver symptoms in 67% of cases, followed by hematological disorders and gastrointestinal pathology. In older children, primary infection leads to liver disease in 51% of cases, with greater neurological and pulmonary involvement compared to that in infants. By highlighting the wide-ranging clinical effects of CMV, we hope to improve physicians’ ability to recognize and subsequently treat this often overlooked condition in pediatric patients.

## 1. Introduction

Cytomegalovirus (CMV), a member of the *Herpesviridae* family, is a ubiquitous virus that commonly infects individuals of all ages, making it one of the most prevalent viral infections worldwide. The global seroprevalence of CMV depends on sex, age, race, ethnicity, socioeconomic status, and education level. Among adult men it varies from 39.3% to 48% [1], and among women of reproductive age, it is estimated at 86% [1], although it varies regionally. In Canada and the United States among women with childbearing potential, it ranges from 25% to 81%, while in Europe, it ranges from 46% to 96%. Additionally, seroprevalence increases with age. In the United States and Canada, 47% of individuals aged 12–20 years are seropositive, while 67–70% of those aged 40–50 years show evidence of prior infection [2].

CMV can be transmitted through direct contact with nearly all body fluids, including saliva, tears, urine, stool, breast milk, and semen from infected individuals [3]. The virus has been shown to remain viable for up to 6 h on surfaces, making transmission via fomites possible. Additionally, CMV can be efficiently transmitted through transplanted organs and blood transfusions [4,5,6,7].

The natural history of CMV infection is complex, involving three distinct types of infection [8]: primary infection, secondary infection or reactivation, and reinfection. Primary infection occurs when an individual who has not been previously immunized is infected for the first time. After primary infection, the virus establishes a latency period in the body. In some cases, the virus can reactivate, resulting in a secondary infection. Additionally, reinfection or superinfection may occur if a person previously infected with CMV is exposed to a different strain of the virus, even if sufficient immunity has developed.

The clinical presentation of CMV infection varies considerably depending on the timing of infection—whether congenital, perinatal, or postnatal—as well as the child’s immune status (primary infection in immunocompromised vs. immunocompetent individuals). Each stage and type of infection can lead to a distinct clinical course.

Congenital CMV infection (cCMV) occurs when the virus is transmitted in utero. Transmission can occur in two ways: through a primary infection in a seronegative woman who acquires CMV during pregnancy or through the reactivation of a latent infection or reinfection with a new CMV strain in a seropositive pregnant woman. Congenital CMV infection is typically defined by a positive CMV test within three weeks after birth. It is a common infection among newborns, with a higher prevalence in low- and middle-income countries compared to that in high-income countries [9]. Around 10–15% of infants with cCMV show symptoms at birth, while the remaining 85–90% are asymptomatic at birth [10].

In seropositive women, perinatal infection can occur when the virus is transmitted to the neonate through cervical–vaginal secretions during delivery. Additionally, breast milk can serve as a source of postnatal CMV infection (pCMV). While pCMV transmission through breast milk is rare and typically asymptomatic in full-term neonates, it can lead to more severe manifestations in preterm infants. This is likely due to their relative lack of maternal antibodies [11]. While numerous studies have explored pCMV infections in preterm infants, there are limited data on full-term infants. Postnatal CMV infection can also occur through direct contact with infected bodily fluids, such as saliva or urine, and is defined by a positive CMV test after the first 3 weeks of life. Most perinatal and postnatal CMV infections are less severe than cCMV infections. The majority of affected infants and children are asymptomatic or experience only mild self-limited symptoms [8].

Immunocompetent children are typically asymptomatic or present with mild symptoms, such as mononucleosis-like syndrome [7]. In immunocompromised children, including those undergoing hematopoietic stem cell transplantation, solid organ transplantation, or receiving chemotherapy, the risk for severe CMV disease is significantly increased. These children are at a higher risk of developing life-threatening complications.

Clinical manifestations, diagnostic approaches, and therapeutic options for CMV infection have been extensively documented in recent years, particularly in immunocompromised children and congenital infections. However, there are limited data on primary CMV infection in immunocompetent children not acquired through transplacental transmission.

This review aims to assess and analyze studies from the last 10 years (2014–2024) regarding the clinical manifestations of postnatal and primary CMV infection in otherwise healthy children.

## 2. Literature Search

### 2.1. Search for Published Cases

A comprehensive search was conducted across four databases—PubMed/MEDLINE, Embase, Scopus, and Web of Science—to identify the relevant literature published between 1 January 2014 and 2 December 2024. Only studies written in English or French were considered for inclusion. The search query included the following keywords: “cytomegalovirus”, “child”, and “immunocompetent”. The selected population ranged from 0 to 17 years of age. Only studies referring to postnatal, acquired, or primary CMV infections were included. Congenital and perinatal infections were excluded. In studies that described both congenital/perinatal and other types of infections, only the non-congenital and non-perinatal infections were considered. In the analyzed paper, all patients underwent tests to confirm a CMV infection. These tests included serological evaluation (detection of IgG and IgM), quantitative PCR on biological fluids (such as blood, urine, saliva, CSF, and bronchoalveolar lavage), and the detection of intracellular CMV in biopsy samples.

### 2.2. Selected Papers

**Figure 1:**
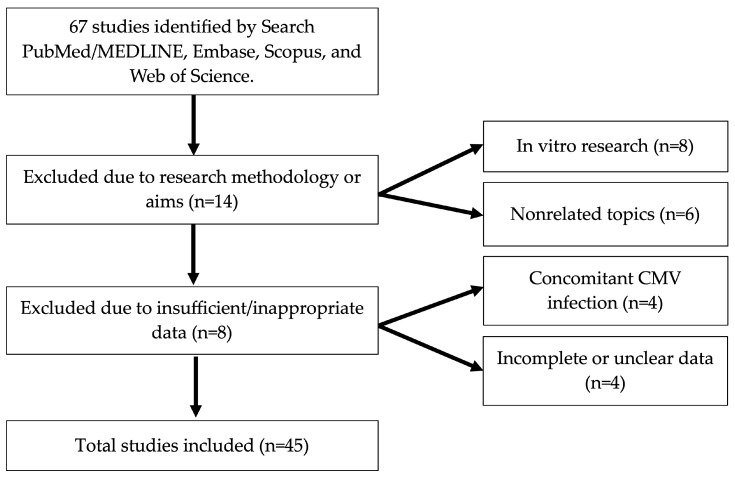
Flow diagram illustrating the identification and inclusion process of studies.

Sixty-seven papers matched the keywords research. Of these, we excluded the following:

- Eight because they reported the results of in vitro research;
- Six because they focused on topics outside our area of interest (CMV effects on T reg activity, CMV-specific T cell immunity, vaccine response and differences in T effector response between immunocompetent children, CMV infection in a child with Down syndrome, radiological findings in pediatric CMV lung infection, and a study of CMV seroprevalence in the Russian adolescent population);
- Four because other pathogens were described as contaminants of CMV infection [12,13,14];
- Four due to incomplete data on CMV infection [15] or no clear data [16,17,18,19] (see Figure 1);
- Therefore, 45 papers were included and are listed in Table 1. The main characteristics of the cases presented are shown.

## 3. Postnatal CMV Infection

The occurrence of symptomatic pCMV infection also shows significant variability. It can occur after exposure to human milk, blood products, or transplanted organs. Postnatal CMV infection in infants typically occurs in the second or third month of life, when there is greater viral shedding in breast milk [65]. Distinguishing between congenital and postnatal CMV infections presents a primary challenge in diagnosing CMV during the perinatal period.

Chen et al. described 251 cases of pCMV but did not distinguish the clinical presentation between term and preterm infants. The most frequent manifestation was hepatitis (150 infants), followed by persistent jaundice (55 infants) and thrombocytopenia (46 infants) [27]. Garozzo et al. did not describe whether the infant with colitis was born full-term or preterm [63].

Regarding thrombocytopenia, although it is more common in congenital CMV, once all other causes have been excluded, CMV should be investigated in both term and preterm infants, as CMV infection can occasionally cause severe and refractory thrombocytopenia.

### 3.1. Preterm Infants

Preterm infants are at higher risk for symptomatic pCMV infection acquired through breast milk due to the relative lack of maternal antibodies. The main risk factors include very low birth weight (<1500 g), low gestational age (<32 weeks), and high levels of CMV DNA in breast milk [66]. The reviewed articles describe a total of 152 cases of pCMV in preterm infants [21,27,29,38,48,53].

Most studies report gastrointestinal involvement, with necrotizing enterocolitis observed in seven infants. One infant had hemophagocytic lymphohistiocytosis [48], and four infants had lung disease [21,29]. Minihan et al. [29], along with other studies [67,68,69,70], also reported hematological disorders (thrombocytopenia, neutropenia), gastrointestinal issues (abdominal distension, hepatomegaly, vomiting), and more severe conditions such as sepsis-like syndrome.

The long-term consequences of pCMV infection remain a subject of debate. A recent systematic review showed that premature infants infected with pCMV have an increased risk of pulmonary and neurological complications, which are associated with poor outcomes [71].

The latest consensus for the management of cCMV infection recommends testing very preterm infants (<32 weeks’ gestation) or very-low-birth-weight infants (<1500 g) at birth for CMV to differentiate between congenital and postnatal infection [72].

### 3.2. Term Infants

Postnatal CMV infection is not typically associated with clinical signs in term infants, likely due to the relative maturity of their immune system and the maternal antibodies acquired during the third trimester of pregnancy [73,74]. Chen et al. [75] found that, in most cases, pCMV infection in term and late preterm (≥32 weeks) infants did not lead to symptoms requiring hospital admission. Since the article did not specifically describe the clinical features of the infants, it was excluded from our analysis. Transmission of the virus, either through vaginal secretions or breast milk, appears to be the cause of infection in these patients.

The analyzed papers reported gastrointestinal involvement in twenty-seven infants [28,35,42,54,58,60,62], hematological involvement (thrombocytopenia and anemia) in nine [37,43], and sepsis-like syndrome only in one [22]. In their paper [54], Sue hypothesized that susceptibility to more severe intestinal infections in some full-term infants may be linked to variations in Toll-like receptors (TLR-2, TLR-4) and nucleotide-binding oligomerization domain-containing protein 2 (NOD2) or in genes encoding similar proteins.

### 3.3. Postnatal CMV Infection and Breast Milk

The primary source of postnatal CMV transmission is through breast milk. This mode of transmission represents the main source for the majority of infants infected during the first year of life [76,77,78]. Maternal immunity or latent infection does not prevent viral shedding due to the possibility of viral reactivation. The timing of viral shedding, its variations, and the cessation can vary significantly between individuals. The exact mechanisms of reactivation are not fully understood. However, it is well-established that CMV DNA undergoes reactivation and can be detected in the breast milk of about 95% of CMV-seropositive mothers [79].

Several single-center studies have utilized prospective surveillance to quantify the risk of acquiring postnatal CMV (pCMV) from breast milk among preterm infants. These studies have shown an association between maternal CMV seropositivity, breastfeeding, and pCMV infection, particularly in extremely low-birth-weight (ELBW) (<1000 g) [66], very-low-birth-weight (<1500 g) [70,80], and extremely preterm infants (<28 weeks) [66,70].

Although breastfeeding is not contraindicated, whether to continue or discontinue providing human milk from a known CMV-seropositive mother to her premature infant remains a subject of debate. This decision must carefully weigh the immune benefits of human milk against the risk of CMV transmission. The only secure method to eliminate viral infectivity is heat inactivation. There are two types of heat inactivation: short-term (62 °C for 5 s) and long-term pasteurization, also known as Holder pasteurization (62.5 °C for 30 min). Short-term inactivation offers the advantages of breastfeeding without the risk of CMV transmission and can be used effectively in routine conditions [81,82]. Holder pasteurization, on the other hand, is the most common procedure for CMV inactivation, but it alters the nutritional and bioactive characteristics of breast milk [78].

Freezing does not completely eliminate the virus; therefore, pasteurization is the preferred method for inactivating CMV in breast milk [83]. Unlike Holder pasteurization, freezing preserves the nutritional and immunological components, enzymes, hormones, growth factors, and CMV-specific antibodies present in human milk [84]. In light of these considerations, when prescribing breast milk or donor human milk for very-low-birth-weight and extremely premature infants, it is important to balance the need to reduce the viral load while preserving as many of the beneficial properties of human milk as possible.

### 3.4. Postnatal CMV Treatment

Regarding treatment, the articles reporting on whether treatment was administered indicated that not all infants received antiviral therapy. Specifically, of the 64 preterm babies affected, 49 were not treated, while 10 received ganciclovir [29,48,53], and 5 received ganciclovir and subsequently valganciclovir [29]. Of the 26 full-term infants, 17 were treated: 14 were given ganciclovir alone [35,42,54,62], 2 valganciclovir alone [28,54], and 1 both ganciclovir and valganciclovir [54]. Although the use of ganciclovir or valganciclovir for pCMV infection in both term and preterm infants has been reported, there are still no large-scale trials focused specifically on the treatment of pCMV-infected infants. The evaluation of potential therapies for pCMV should consider factors such as the severity of the clinical condition caused by CMV infection, the side effects of the treatment, any underlying conditions that may predispose to or exacerbate infection, and the viral load.

## 4. Primary Infection in Childhood

While CMV is a well-recognized pathogen in neonates, immunocompromised children, and adults, the burden of CMV disease in immunocompetent children is less well-described. Most people contract the virus at some point in their lives. CMV can be found in oropharyngeal secretions, semen, tears, urine, feces, and blood, in addition to vaginal and cervical secretions (the cause of perinatal CMV infection) or breast milk (the main cause of pCMV infection). Intimate contact is necessary for the horizontal transmission of CMV. Symptomatic CMV infection is significantly increased in immunocompromised patients.

Primary infection often occurs during childhood or adolescence. In a prospective cohort study of adolescent girls in the USA, Finland, and Mexico, who were followed for three years, the baseline CMV seroprevalence was 58%. Primary infection occurred in 14.8% of seronegative girls. Among the seropositive girls, 5.9% had an at least four-fold increase in anti-CMV antibodies, and 23.9% excreted CMV DNA in urine [10].

In a recent review, various clinical manifestations associated with CMV infection in immunocompetent adults were described, including gastrointestinal, central nervous system, ocular, pulmonary, hematological, cardiac, and skin involvement [85]. Even in the immunocompetent pediatric population, infection can present with a variety of clinical manifestations, as described in detail below.

### 4.1. Gastrointestinal Disease and Hepatitis

As found in adults [85], our analysis shows that in immunocompetent patients, gastrointestinal symptoms are closely associated with CMV infection. The liver is the most commonly affected organ, with 137 cases of hepatitis described in the studies we analyzed [30,50,51]. In a large, decade-long case series from Korea [50], differences in liver signs and symptoms were observed in patients under and over three months of age. Specifically, jaundice was the primary symptom in patients under three months of age. Regardless of age, elevated aminotransferases and fever were present in all patients.

Digestive tract involvement was described in a smaller number of cases. Eight cases with colon involvement were described in patients without inflammatory bowel disease [34], twenty-two with protein-losing enteropathy [25,40], one with gastritis [26], and one with appendicitis [20]. In most cases (25/32, 78.1%), inclusion bodies with tissue positive for CMV DNA polymerase chain reaction (PCR) were found in the affected gastrointestinal segment. Altered CMV serology or detection of CMV in blood or other bodily fluids was observed in the remaining patients. No specific symptoms were described for gastrointestinal involvement. However, if other diagnoses are excluded, CMV infection should be considered in patients with prolonged diarrhea, peripheral edema, bloody stools, and abdominal pain.

### 4.2. Respiratory Disease

The literature contains a small number of publications on CMV respiratory tract infection in immunocompetent infants. Cinel et al., in a study involving 102 children with persistent wheezing [59], demonstrated that the most reliable way to diagnose lower respiratory tract infections is by detecting CMV in bronchoalveolar lavage fluid. Bronchoalveolar lavage fluid CMV PCR was found to be superior to blood CMV PCR in diagnosing lower respiratory tract infections caused by CMV in immunocompetent infants. Along with wheezing, the patients in this study also presented with rhonchi, tachypnea, retractions, and hypoxia. Radiological studies revealed interstitial infiltration and atelectasis, with ground-glass opacities on CT images. Another paper [49] described a severe case of CMV pneumonia that progressed to multiple organ dysfunction syndrome, with hypotension, acute renal failure, liver failure, and disseminated intravascular coagulation. The authors suggested that the clinical worsening was likely due to a delay in diagnosis, as the CMV test was only performed at a later stage of the disease. A recent study of the Chinese pediatric population [86], excluded from our analysis because the data did not specify how many patients were exclusively infected with CMV versus those with mixed infections, showed a high incidence of acute respiratory CMV infection (24.49% of 15,993 children aged 28 days to 14 years). Despite this limitation, this large population-based study indicated that respiratory CMV (whether co-infected or not) was common. Therefore, CMV should always be included in the proposed test panel, along with other pathogens.

### 4.3. Hematological Diseases

Thrombocytopenia can occur with all viral infections, but very little is known about platelet counts during primary CMV infection in immunocompetent hosts, as CMV infection is often unrecognized due to its asymptomatic nature. Immune thrombocytopenic purpura is a form of acquired thrombocytopenia triggered by anti-platelet antibodies, which peripherally destroy platelets, damage megakaryocytes, and inhibit platelet production in the marrow [87,88]. CMV has been identified as one of the causes of this form of thrombocytopenia (CMV-related secondary immune thrombocytopenia). Alternatively, CMV infection can directly affect megakaryocytes, leading to thrombocytopenia (CMV-induced thrombocytopenia). Distinguishing between CMV-associated and CMV-induced thrombocytopenia can be challenging; therefore, these patients are often referred to as having CMV-associated thrombocytopenia. In the cases described, three patients had CMV-related secondary immune thrombocytopenia [45], while twelve children had CMV-induced thrombocytopenia [44,45,46].

The onset of autoimmune hemolytic anemia in CMV-infected patients may be triggered by a similar immune response [36,47].

### 4.4. Brain Diseases

Central nervous system involvement in CMV infection is of particular concern in congenital infections, especially if contracted during the first trimester of pregnancy [72]. Brain involvement in non-congenital infections has primarily been described in immunocompromised individuals, such as those with HIV/AIDS, those undergoing immunosuppressive therapy, or those post-organ transplantation, as well as in the course of severe hematological diseases. Three studies were included in our selection: one describing 18 cases of encephalitis identified in a 15-year retrospective study at a hospital in China [41] and two describing cases of acute disseminated encephalomyelitis [32,33].

Guo and Jiang [41] found that CMV encephalitis was predominantly observed in infants aged 4–6 months. In this study, 95% of patients presented with convulsions at symptom onset, 78% with fever and poor appetite, and approximately half experienced altered consciousness. Ganciclovir treatment led to symptom improvement within 3 to 10 days. At follow-up, half of the patients showed no sequelae, 16% experienced recurrent convulsions, and 11% had delayed psychomotor development. The severity of sequelae appeared to correlate with the genomic viral load in urine and cerebrospinal fluid (CSF). The disappearance of passive immunity due to the loss of maternally acquired CMV-specific antibodies may explain the high proportion of symptomatic CMV infections during this period. However, the status of immune memory to CMV in infants at this age remains unclear.

In cases of encephalomyelitis, patients presented with progressive, symmetric weakness [32], or with ataxia, altered behavior, or vertical nystagmus [31]. Both studies noted improvement following corticosteroid administration. It is believed that CMV triggers an autoimmune response against specific myelin-like antigens in these cases. This molecular mimicry can lead to T cell activation, inflammation, and subsequent demyelination [89,90].

### 4.5. Vascular Damage

CMV infection and vascular damage are not clearly correlated, particularly in immunocompetent hosts. While cases of CMV infection directly causing vascular damage have been described [91], immune-mediated vascular damage resulting from the activation of an autoimmune response has also been reported [92]. In the cases we reviewed, there was no involvement of specific vascular districts. However, one case of cutaneous vascular involvement in a three-year-old boy [61] and one case of transient cerebral arteriopathy in a two-year-old girl [55] were included.

### 4.6. Adrenal Disease

Adrenal involvement in patients without immunological deficits is a rare occurrence. In the decade under review, only one case was described involving a 10-week-old infant, who developed an adrenal crisis following a short febrile illness and severe prolonged watery diarrhea, accompanied by hypertransaminasemia and a rash [31]. Adrenal gland involvement during CMV infection has been predominantly reported in patients with HIV. In these patients, immune deficiency, characterized by insufficient CD4-dependent cytotoxic T lymphocyte activity and reduced CMV antigen-specific CD8 + T cell responses, leads to an inadequate immune response to CMV. At the adrenal level, CMV may directly invade the adrenal glands and form CMV inclusions within the organ [93,94].

### 4.7. Other Conditions

CMV infection can cause other non-specific symptoms that are unlikely to be CMV-related, such as prolonged fever (one case) [24], mononucleosis-like illnesses (three cases) [23,52,57], sepsis with hepatitis and autoimmune hemolytic anemia (one case) [36], and hemorrhagic cystitis (one case) [64].

CMV is estimated to cause 7% of mononucleosis-like illnesses. Children and adolescents in close contact with infected individuals, especially those under the age of two, are at a higher risk of acute CMV infection. Although primary infection is usually asymptomatic, CMV can cause mononucleosis-like illness, which is difficult to clinically distinguish from Epstein–Barr virus (EBV) mononucleosis [95]. The symptoms of CMV and EBV infections are very similar, including fatigue, fever, hepatosplenomegaly, lymphadenopathy, and pharyngitis. The key difference is that transaminases are more frequently elevated in cases of CMV infection [96]. Mononucleosis-like illnesses can also cause severe disease with multi-organ involvement, such as hepatopathy, anemia, thrombocytopenia, proteinuria, pleural effusion, and ascites [23]. In all cases of patients with mononucleosis-like syndrome, CMV should be considered alongside or after other serological tests. Rodrigues et al. also tested for EBV, *Leptospira*, *Rickettsia*, *Leishmania*, and hepatitis A–E [23], while Raja et al. tested for EBV and Influenza A and B [57].

A similar clinical condition, poorly described in children, is lymphoma-like syndrome. In the four patients described by Viguè et al. [56], non-specific signs and symptoms were present, including prolonged fever, hepatosplenomegaly, lymphadenopathy, and abdominal pain.

**Figure 2:**
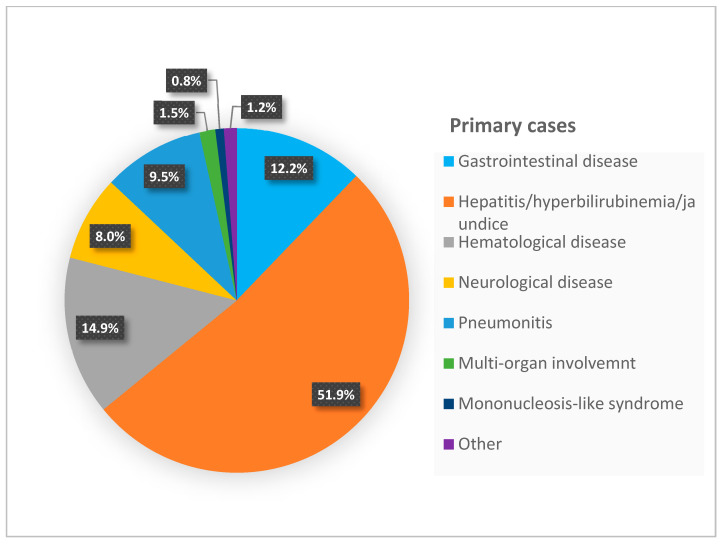
Graphical representation of symptoms in postnatal cases.

**Figure 3:**
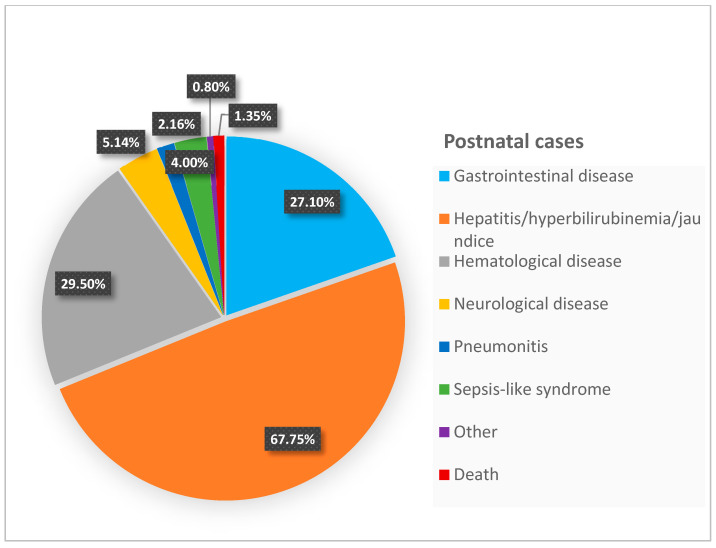
Graphical representation of symptoms in primary cases.

In Table 2, we categorized the systems involved, while Figure 2 and Figure 3 graphically represent the symptoms in both postnatal and acquired infections. This classification may aid in the early detection and timely intervention of CMV infection, which is crucial for improving clinical outcomes. Among the 369 cases of pCMV, more than half presented with hepatic symptoms, and approximately 30% exhibited hematological symptoms (primarily thrombocytopenia), with another 30% presenting with gastrointestinal symptoms. In the 262 cases of primary infection, around half had hepatic involvement. Neurological symptoms not typically seen in pCMV were reported in this group. No patients with primary CMV infection died. It is likely that the deaths among pCMV patients were not directly and exclusively attributed to the viral infection.

The diagnosis of CMV infection can be made through serologic assays, molecular testing, or viral isolation via conventional cell culture. In some cases, a tissue biopsy may also be utilized for confirmation.

Several serologic tests are available to detect both IgG and IgM antibodies specific to CMV. To diagnose a recent infection, testing for CMV IgG in paired serum samples collected at least two weeks apart, alongside IgM testing in a single serum sample, can be helpful. Low-avidity CMV IgG in the presence of CMV IgM suggests a recent infection, while high-avidity IgG indicates a past infection.

Viral DNA can be detected using polymerase chain reaction (PCR) and other nucleic acid amplification techniques in various tissues and fluids. However, detecting CMV DNA in blood via PCR does not necessarily indicate an active infection, especially in individuals with a competent immune system.

CMV can also be isolated through conventional cell culture from urine, saliva, peripheral blood leukocytes, human milk, semen, cervical secretions, and other tissues and fluids. Isolation from a target organ strongly supports CMV infection as the cause of the disease. Standard viral cultures should be maintained for at least 28 days before being considered negative, although this method is not commonly used in routine diagnostics due to its time-consuming nature.

In biopsy tissue, CMV infection can be diagnosed based on the presence of characteristic “owl’s eye” inclusions on histological examination, which are pathognomonic of CMV infection. These inclusions are typically found in the nuclei of infected cells, and their presence can strongly indicate CMV. Additionally, immunohistochemical (IHC) staining can be used to detect CMV-specific antigens within tissue samples. IHC is particularly valuable in detecting viral proteins in tissues with low viral loads or subtle histological changes [97].

Table 3 lists the diagnostic investigations for suspected pCMV or primary infection in immunocompetent pediatric patients included in the analysis.

### 4.8. Therapy in Immunocompetent Children

Most current studies focus on therapy for immunocompromised individuals, where foscarnet and cidofovir can be considered in addition to ganciclovir and valganciclovir [98]. In these patients, drugs that target resistant CMV, such as miravabir, have also recently been approved [93,94].

There are no specific guidelines according to which immunocompetent children should be treated. Avila-Agüero et al. demonstrated in their study that the use of ganciclovir in immunocompetent pediatric patients was associated with negative antigenemia in 80% of treated patients [99]. The studies analyzed show that, even within the same study group or for similar diseases, there is no clear consensus on treatment. For example, in Cinel’s study, not all patients were treated (14/25 wheezing infants were treated with ganciclovir) [55]. In Gou’s study, 11 out of 18 patients with encephalitis were treated with ganciclovir [37]. In Jin’s study, 8 out of 11 patients with thrombocytopenia were treated with ganciclovir only or both ganciclovir and valganciclovir [41]. Similar findings are seen when comparing different articles on the same pathology. For instance, in patients with encephalomyelitis, one study reports treatment with ganciclovir [29], while another does not [35]. Similarly, in patients with mononucleosis-like illness, one study reports treatment with ganciclovir [48], while another does not [53]. A high-quality clinical trial is needed to assess the efficacy and safety of antiviral therapy in immunocompetent patients with severe CMV disease.

Regarding the administration of immunoglobulins (IVIG) and/or steroids, treatment depends on the clinical presentation. In eight studies [20,27,29,30,55,58,60,61], steroids were administered before the diagnosis of CMV infection based on suspicion of other diseases such as Crohn’s disease, Henoch–Schönlein syndrome, or following clinical deterioration. In other cases, steroids were indicated for the treatment of acute demyelinating encephalomyelitis [31,32] or for conditions such as warm-reactive autoimmune hemolytic anemia [35,42,46], often in combination with IVIG if there was only a partial response to steroids. In several cases of CMV-associated thrombocytopenia, the clinical condition resolved after IVIG administration, similar to immune thrombocytopenic purpura (ITP) [100]. However, CMV-associated thrombocytopenia may be less responsive to standard ITP therapies, including IVIG and steroids, and may require antiviral treatment [101]. The response to IVIG and steroids depends on the underlying mechanisms of CMV-induced thrombocytopenia [102,103]. CMV can cause immune dysregulation and platelet destruction through molecular mimicry by viral antigens, leading to the production of anti-platelet antibodies [88,104], or it can directly infect megakaryocytes, causing maturation arrest and resulting in decreased platelet production [105]. The latter mechanism better explains the lack of response to conventional ITP therapies, as these treatments are aimed at preventing platelet destruction rather than enhancing platelet production [101].

## 5. Conclusions

CMV is a versatile pathogen responsible for a wide spectrum of clinical manifestations, ranging from mild symptoms to severe, life-threatening complications. The most well-described clinical presentations of CMV infection are typically associated with congenital cases and those affecting immunocompromised infants and children. However, the impact of the virus extends far beyond these groups, with significant disease potential even in postnatal or acquired infections in immunocompetent children.

The lack of large-scale studies in this area is likely due to the fact that postnatal or acquired CMV infections are often unrecognized or diagnosed late. This is primarily because CMV infections in immunocompetent hosts tend to be asymptomatic or exhibit only mild symptoms with a self-limited course. As a result, clinicians may frequently overlook CMV as a potential cause of severe disease in otherwise healthy patients.

Given the ability of CMV to affect multiple organ systems, it is crucial to recognize it as a cause of severe infection, even in immunocompetent individuals. The clinical severity of CMV infections can vary significantly, necessitating a range of treatment strategies. These strategies may include symptomatic therapies, immunoglobulins, corticosteroids, and antiviral treatments, depending on the complexity of the case.

Regarding prevention, the only viable option for preventing CMV infection is available to premature and term infants with specific pathological conditions who are still hospitalized. In these patients, heat inactivation of breast milk prior to administration may be considered a preventive measure against postnatal CMV infection. In order to achieve timely diagnosis and appropriate treatment, healthcare providers must remain vigilant in recognizing the diverse and occasionally severe presentations of CMV infection, even in cases not associated with a known immunodeficiency.

## Figures and Tables

**Table 1:** List of papers included published from 2014 to 2024. For each study, the following factors were considered: the clinical manifestations described, the age of the patients (including median age if available), the type of infection (postnatal or primary), the use of antiviral drugs targeting CMV (ganciclovir and/or valganciclovir), and the administration of other medications (such as corticosteroids and/or intravenous immunoglobulins).

Author, Year	Clinical Manifestation (*n* Cases)	Age (Median Age)	Type of Infection (*n* Cases)	Antiviral Therapy (*n* Cases)	Other Therapy (*n* Cases)
Bedell M, 2024 [20]	Appendicitis (1)	7 years old	Primary	No	No
Aboelsoud K, 2024 [21]	Pneumonitis, thrombocytopenia (1)	Born at 25 weeks of gestational age	Postnatal	No	Corticosteroids
Salemi T, 2024 [22]	Sepsis-like syndrome (1)	39 days old	Postnatal	Ganciclovir	No
Rodrigues A, 2024 [23]	Disseminate infection and multi-organ involvement (1)	7 years old	Primary	Ganciclovir and valganciclovir	No
Eid M, 2023 [24]	Prolonged fever (1)	11 years old	Primary	Ganciclovir	No
Ferrua C, 2023 [25]	Protein-losing enteropathy (21)	1–133.7 months old (median 29.7 months)	Primary	Antiviral (5) (drug not specified)	Immunoglobulin (2) Corticosteroids (2)
Semwal P, 2023 [26]	Gastritis and gastric outlet obstruction (1)	4 years old	Primary	Ganciclovir and valganciclovir	No
Chen YN, 2023 [27]	251 cases: - hepatitis (150) - prolonged jaundice (55) - thrombocytopenia (46) - neurological involvement (19) - pneumonitis (4) - other (10)	<90th day of life (140 cases) >90th day of life (111 cases)	Postnatal	Ganciclovir (32)	No
Cascardo C, 2022 [28]	Colitis (1)	140 days old	Postnatal	Valganciclovir	Corticosteroids
Minihan L, 2022 [29]	Total preterm (48) (<32 weeks and/or <1500 g) - hyperbilirubinemia/jaundice (15) - hepatomegaly/elevated liver enzymes (29) - thrombocytopenia/petechiae (32) - neutropenia (23) - sepsis-like syndrome (14) - abdominal distention/vomiting/altered stool (40) - pneumonitis (3) - death (2)	47–82 days old	Postnatal	Ganciclovir (6) Ganciclovir and valganciclovir (5)	No
Wagoner M, 2022 [30]	Hepatitis and hemophagocytic lymphohistiocytosis (1)	18 months old	Primary	No	Corticosteroids
Fuchs S, 2022 [31]	Adrenal injury (1)	10 weeks old	Primary	Ganciclovir	Corticosteroids
Wan Natrah WY, 2022 [32]	Acute disseminated encephalomyelitis (1)	11 years old	Primary	No	Corticosteroids
Da Silva RC, 2021 [33]	Acute disseminated encephalomyelitis (1)	17 years old	Primary	Acyclovir	Corticosteroids
Bayrak N, 2021 [34]	Colitis (8)	2–23 months old (9.8 months)	Primary	Ganciclovir (4) Ganciclovir + valganciclovir (2)	No
Howard-Jones AR, 2021 [35]	Enterocolitis, hepatitis, and thrombocytopenia (1)	10 weeks old	Postnatal	Ganciclovir	No
Choudhary A, 2021 [36]	Sepsis, progressive hepatitis, and autoimmune hemolytic anemia (1)	11 months old	Primary	Ganciclovir	Immunoglobulin
Hu H, 2021 [37]	Thrombocytopenia (8)	<12 months old	Postnatal	No	Corticosteroids (8) Immunoglobulin (6)
Patel RM, 2020 [38]	Total CMV + (33) necrotizing enterocolitis (6) death (3)	NA (mean GA: 27.9 weeks)	Postnatal	Not specified	Not specified
Wang Y, 2020 [39]	Colitis (8)	2–6.3 months old (2.5 months)	Postnatal	Ganciclovir (6)	No
Tuna Kirsaclioglu C, 2020 [40]	Protein-losing gastropathy (1)	5 years old	Primary	No	No
Guo Y, 2019 [41]	Encephalitis (18)	37–790 days old (5.1 months)	Primary	Ganciclovir (11)	No
Goh J, 2019 [42]	Enterocolitis (1)	4 months old	Postnatal	Ganciclovir	No
Loureiro B, 2019 [43]	Autoimmune hemolytic anemia (1)	6 months old	Postnatal	No	Corticosteroids Immunoglobulin
Nishio Y, 2018 [44]	Thrombocytopenia (1)	20 months old	Primary	No	Immunoglobulin
Jin MJ, 2018 [45]	Thrombocytopenia (29) CMV-induced thrombocytopenia (23) CMV-related secondary immune thrombocytopenia (6)	>6 months old	Primary	Ganciclovir or Ganciclovir + valganciclovir (8)	Immunoglobulin (13) Corticosteroids (4) Eight patients underwent combined therapy
Isleyen F, 2018 [46]	CMV-related secondary immune thrombocytopenia (3)	2.5–11 months old	Primary	No	Immunoglobulin (3)
Khalifeh HK, 2017 [47]	Autoimmune hemolytic anemia (1)	6 months old	Primary	No	Immunoglobulin Corticosteroids
Silwedel C, 2017 [48]	Hemophagocytic lymphohistiocytosis (1)	9 weeks old (born late preterm)	Postnatal	Ganciclovir	No
Al-Eyadhy AA, 2017 [49]	Pneumonia, multi-organ failure (1)	12 years old	Primary	Ganciclovir	No
Min CY, 2017 [50]	Hepatitis (123)	14 days–11.3 months old (8.5 months)	Primary	No	No
Tsunoda T, 2017 [51]	Hepatitis (13)	1–12 months old (7 months)	Primary	Not specified	Not specified
Alvarez-Hernadez L, 2017 [52]	Mononucleosis-like syndrome (1)	5 years old	Primary	Ganciclovir	No
Goelz R, 2016 [53]	Abdominal distension, bloody diarrhea, NEC with perforation, and volvulus with intestinal necrosis (15) * Diarrhea (2) **	NA *: preterm 23–25 weeks **: term	Postnatal	Ganciclovir (5)	No
Sue P, 2016 [54]	Invasive enterocolitis (21)	21 days old–14 months old (2 months)	Postnatal	Ganciclovir (11) Ganciclovir and valganciclovir (1) Valganciclovir (1) No (8)	No
Kao W, 2015 [55]	Transient cerebral arteriopathy (1)	2.5 years old	Primary	Ganciclovir	Immunoglobulin Corticosteroids
Viguè MG, 2015 [56]	Lymphoma-like syndrome (4)	33–82 months old	Primary	No	Corticosteroids (1)
Raja J, 2015 [57]	Mononucleosis-like syndrome (1)	12 years old	Primary	No	No
Marseglia L, 2014 [58]	Colonic stenosis post NEC (1)	Newborn	Postnatal	Ganciclovir	No
Cinel G, 2014 [59]	Pneumonitis (25)	4–30 months old (11.3 months)	Primary	Ganciclovir (14)	Corticosteroids (1)
Louazon T, 2014 [60]	Colitis (1)	10 weeks old	Postnatal	No	No
D’Alessandro M, 2014 [61]	Necrotizing vasculitis mimicking Henoch–Schönlein syndrome (1)	3 years old	Primary	No	Corticosteroids
Novakova V, 2014 [62]	Severe colitis (1)	2 months old	Postnatal	Ganciclovir	Corticosteroids Immunoglobulin
Garozzo MT, 2014 [63]	Colitis (1)	8 weeks old	Postnatal	Ganciclovir	No
Taktak A, 2014 [64]	Hemorrhagic renal cystitis (1)	3 years old	Primary	No	No

**Table 2:** Number of patients with system-specific symptoms. The “Other” category included conditions such as prolonged fever [24], failure to thrive, myocarditis, nephrotic syndrome [27], adrenal injury [31], and hemorrhagic cystitis [64]. From the 33 CMV-positive patients described in Patel’s article [37], we included only the nine symptomatic patients. * In the case series by Chen [27] and Minihan [29], different patients exhibited more than one sign or symptom. Therefore, we calculated the total number of occurrences for each manifestation.

	Manifestations in 369 Postnatal Cases * Number (%)	Manifestations in 262 Primary Cases Number (%)
Gastrointestinal disease	100 (27.1%)	32 (12.2%)
Hepatitis/hyperbilirubinemia/jaundice	250 (67.75%)	136 (51.9%)
Hematological disease	109 (29.5%)	39 (14.9%)
Pneumonitis	8 (2.16%)	25 (9.5%)
Neurological disease	19 (5.14%)	21 (8%)
Multi-organ involvement	-----	4 (1.5%)
Mononucleosis-like syndrome	-----	2 (0.8%)
Sepsis-like syndrome	15 (4%)	---
Other	3 (0.8%)	3 (1.2%)
Death	5 (1.35%)	---

**Table 3:** Diagnostic investigations.

**Exclusion of other infectious diseases**	EBV, HSV, Hepatitis, Influenza, bacterial infection, other related to pathology
**Serological evaluation**	IgG and IgM, IgG avidity index
**Quantitative PCR on biological fluids**	Urine, saliva, blood Bronchoalveolar lavage (in pulmonary infection) CSF (in neurological desease)
**CMV search in biopsy samples related to pathology (i.e., gastrointestinal tract, bone marrow…)**	“Owl eye” inclusion bodies on histological examination Positive immunohistochemistry for CMV

## Data Availability

No new data were created or analyzed in this study. Data sharing is not applicable to this article.
